# First evidence of Zika virus venereal transmission in *Aedes aegypti* mosquitoes

**DOI:** 10.1590/0074-02760170329

**Published:** 2017-11-27

**Authors:** Jordam William Pereira-Silva, Valdinete Alves do Nascimento, Heliana Christy Matos Belchior, Jéssica Feijó Almeida, Felipe Arley Costa Pessoa, Felipe Gomes Naveca, Claudia María Ríos-Velásquez

**Affiliations:** 1 Fundação Oswaldo Cruz-Fiocruz, Instituto Leônidas e Maria Deane, Laboratório de Ecologia de Doenças Transmissíveis na Amazônia, Manaus, AM, Brasil; 2 Fundação Oswaldo Cruz-Fiocruz, Instituto Leônidas e Maria Deane, Programa de Pós-Graduação em Condições de Vida e Situações de Saúde na Amazônia, Manaus, AM, Brasil

**Keywords:** Zika virus, sexual transmission, mosquito vectors, mating, Flavivirus

## Abstract

**BACKGROUND:**

*Aedes aegypti* is considered the main Zika virus (ZIKV) vector, and is thought to be responsible for the 2015-2016 outbreak in Brazil. Zika positive *Ae. aegypti* males collected in the field suggest that vertical and/or venereal transmission of ZIKV may occur.

**OBJECTIVES:**

In this study, we aimed to demonstrate that venereal transmission of ZIKV by *Ae. aegypti* can occur under laboratory conditions.

**METHODS:**

*Ae. aegypti* collected in the city of Manaus, confirmed as negative for Zika, Dengue and Chikungunya virus by reverse transcription real-time polymerase chain reaction (RT-qPCR) (AaM3V^-^ strain), were reared under laboratory conditions and used for the experiments. The ZIKV used in this study was isolated from a patient presenting with symptoms; ZIKV was confirmed by RT-qPCR. Experiment 1: virgin male mosquitoes of AaM3V^-^ strain were intrathoracically inoculated with a ZIKV suspension; four days after injection, they were transferred to a cage containing virgin females of AaM3V^-^ strain and left to copulate for five days. Experiment 2: virgin female mosquitoes of AaM3V^-^ strain were orally infected with a ZIKV suspension by blood feeding membrane assay; nine days after blood feeding, they were placed in cages with *Ae. aegypti* AaM3V^-^ virgin males and left to copulate for four days. After copulation, all mosquitoes were individually evaluated for viral infection by RT-qPCR.

**FINDINGS:**

The mean infection rate in Experiment 1 and Experiment 2 was 45% and 35%, respectively. In both experiments, cycle threshold values ranged from 13 to 35, indicating the presence of viral genomes.

**MAIN CONCLUSION:**

*Ae. aegypti* males intrathoracically inoculated with a ZIKV suspension are infected and can transmit the virus to uninfected females by mating. Moreover, *Ae. aegypti* females orally infected with a ZIKV suspension can transmit the virus to uninfected males by copulation. This study shows that ZIKV infection of *Ae. aegypti* mosquitoes occurs not only during blood feeding, but also during copulation.

Zika is a disease caused by an arbovirus (Zika virus, or ZIKV) of the *Flaviviridae* family, *Flavivirus* genus. It is a worldwide public health concern. ZIKV was first described in Africa in 1947 ( Mukwaya & Sempala 1977 ), and for a long time, it was thought to cause only a benign illness. However, after its emergence in Brazil in 2015 and its spread throughout most of Latin America and the Caribbean, ZIKV infection has been associated with thousands of cases involving severe complications such as microcephaly, Guillain-Barré syndrome, and death. This severe and unexpected epidemic led the World Health Organization (WHO) to recognise ZIKV as a Public Health Emergency of International Concern ( Azevedo et al. 2016 , Santos et al. 2016) . Zika symptoms are mild and characterised by the sudden onset of fever, maculopapular rash, arthralgia, myalgia, headache, nonpurulent conjunctivitis, pruritus, joint oedema, and exanthema ( Azevedo et al. 2016 , Nunes et al. 2016 , Vasconcelos & Calisher 2016) . Between 2015 and 2016, a total of 707,133 cases were recorded across 48 countries ( Ikejezie et al. 2017) . In 2016 alone, 205,578 cases and eight deaths were recorded in Brazil. The highest incidence rates in Brazil have been recorded in the Central-West Region (231 cases per 100,000) and the North Region (157 cases per 100,000). As of June 2017, there have been 13,353 confirmed cases of Zika, and 322 cases of microcephaly associated with congenital Zika virus infection ( MS/SVS 2017) .

ZIKV has been found infecting the salivary glands of several species of mosquitoes from the *Aedes* and *Culex* genera, but the infection susceptibility of a particular species seems to be strongly associated with specific virus strains ( Fernandes et al. 2016 , Ferreira-de-Brito et al. 2016 , Guedes et al. 2017 ). *Ae. aegypti* is considered the main ZIKV vector (WHO 2016a) and is thought to be responsible for the 2015-2016 outbreak in Brazil ( Oliveira et al. 2016 ). Moreover, ZIKV can infect and be transmitted by American populations of *Ae. aegypti* and *Ae. albopictus* ( Chouin-Carneiro et al. 2016 , Costa-da-Silva et al. 2017 ).

In addition to transmission by mosquito bite, ZIKV can be sexually transmitted between humans; ZIKV has been detected in human semen ( Musso et al. 2015 ). In monkeys, ZIKV can generate viraemia sufficient to infect competent mosquito vectors when introduced intrarectally or intravaginally ( Musso et al. 2015 , Haddow et al. 2017 , Hastings & Fikrig 2017 ). Zika-positive *Ae. aegypti* males have been collected in the field which suggests that vertical and/or venereal transmission of ZIKV may occur between mosquitoes ( Ferreira-de-Brito et al. 2016 , Thangamani et al. 2016) . The maintenance of arboviruses in nature is greatly enhanced when venereal transmission occurs in conjunction with other transmission mechanisms. Venereal transmission has been demonstrated in *Ae. aegypti* infected with the Chikungunya virus ( Mavale et al. 2010) .

This study shows that venereal transmission of ZIKV by *Ae. aegypti* can occur under controlled laboratory conditions. This is an important finding because it may partially explain the high dispersion rate of infected mosquitoes during Zika epidemics, and it highlights the importance of mosquito control programs.

## MATERIALS AND METHODS

*Mosquitoes* - Larvae and pupae of *Ae. aegypti* mosquitoes were collected in January, 2016 from water tanks and discarded containers located around households in Nova Cidade and Adrianópolis neighbourhoods, around the city of Manaus, Amazonas state, Brazil. Field-collected specimens were reared through two generations under laboratory conditions. Individual F2 adult females were stored at -80ºC and the individual batch egg / female were separated and kept for breeding and assays. Total RNA from each F2 female was extracted with TRIzol^®^ Reagent (Thermo Fisher, Waltham, MA) following manufacturer’s instructions, and tested for the presence of ZIKV ( Lanciotti et al. 2008 ), CHIKV ( Lanciotti et al. 2007 ) and DENV ( Gurukumar et al. 2009 ) by reverse transcription real-time polymerase chain reaction (RT-qPCR). Batch eggs from F2 individual adult females that tested negative for all three viruses were used to establish the virus-free mosquito colony, named AaM3V^-^. The mosquito colony was maintained under laboratory conditions at 27ºC with 70% relative humidity. Larvae were reared in plastic containers containing tap water and were fed with fish food. Adults were kept in plastic cages and offered a 10% sucrose solution *ad libitum* .

*ZIKV strain* - The ZIKV used in this study was isolated from a female patient presenting with classical symptoms of arbovirus infection. Zika infection was confirmed by RT-qPCR ( Lanciotti et al. 2008 ). This strain was obtained after the second passage on *Ae. albopictus* C6/36 cells kept at 28ºC in Leibovitz’s L-15 medium supplemented with 2% foetal bovine serum (FBS) and an antibiotic/antimycotic.

After three days of infection on C6/36 cells, viral titre was determined by flow cytometry, based on a previously published protocol for the Dengue virus ( Medina et al. 2012 ). Briefly, we used the anti-flavivirus 4G2 monoclonal antibody, followed by anti-mouse IgG Alexa 488-conjugated secondary antibody, diluted at 1:2,000 and 1:3,000, respectively. After incubation and washing, cells were analysed by flow cytometry, counting at least 100,000 events.

**Fig. 1:**
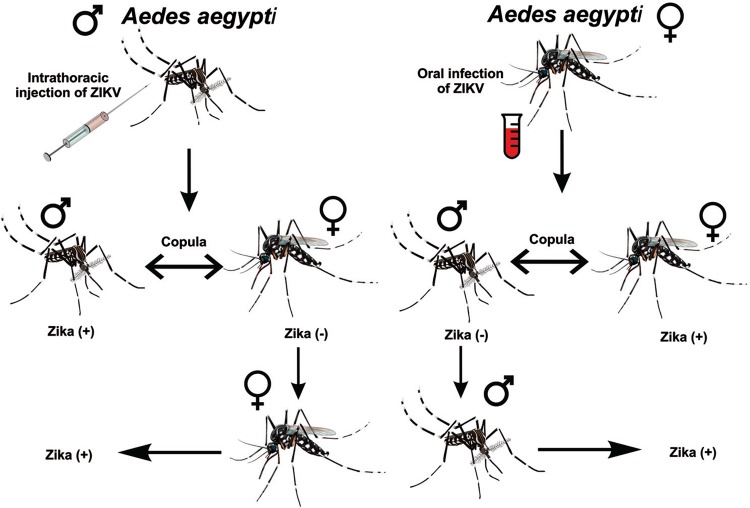
: Zika virus (ZIKV) venereal transmission in *Aedes aegypti* mosquitoes. (A) ZIKV-infected males mating with ZIKV-free females; (B) ZIKV-infected females mating with ZIKV-free males.

*Intrathoracic microinjection of mosquitoes* - One group containing 50 (2-day-old) male mosquitoes was intrathoracically inoculated with a 0.2-µL suspension of ZIKV at a titre of 10^7^ infectious units/mL (FACS IU/mL), following Rosen and Gubler (1974) . Microinjection was made using the Nanoject II injector (Drummond Scientific Company). Mosquitoes were subsequently maintained at 27ºC with 70% relative humidity, and offered a 10% sucrose solution *ad libitum* . Four days after injection, the male mosquitoes were transferred to a cage containing 100 (6-day-old) virgin females and left to copulate for five days at a proportion of one male to two females. After the 5-day copulation period, all females were individually placed in 1 mL of TRIzol^®^ Reagent and stored at -80ºC prior to viral detection by RT-qPCR ( Fig. 1A ). Two independent biological experimental replicates were made for this assay.

*Oral infection of mosquitoes* - One group containing 50 (2-day-old) virgin females was fed using a Parafilm^®^ membrane. A suspension of ZIKV at a titre of 10^7^ infectious units/mL (FACS IU/mL) was used for blood feeding. Fully engorged females were transferred to plastic cages and maintained at 27ºC with 70% relative humidity, and offered a 10% sucrose solution *ad libitum* . Nine days after blood feeding, the females were placed in cages with *Ae. aegypti* AaM3V^-^ virgin 2-day-old males and left to copulate for four days at a proportion of one male to two females. After the 4-day copulation period, 20 surviving males were placed individually in 1 mL of TRIzol^®^ Reagent and stored at -80ºC ( Fig. 1B ). Two experimental biological replicates were made.

Four days postinfection (dpi) was judged to be sufficient time for ZIKV to disseminate throughout the body and into the seminal fluid of the male mosquitoes, and 9 dpi was judged to be sufficient time for the arbovirus to be transmitted venereally and be detectable in the female mosquitoes.

*ZIKV detection in mosquitoes* - Mosquitoes were individually homogenised in 1 mL of TRIzol^®^ Reagent and RNA was extracted following manufacturer’s instructions. Viral RNA was detected using TaqMan^®^ Fast Virus 1-Step Master Mix in a StepOnePlus Real Time PCR System (Applied Biosystems) using ZIKV primers and probes described previously ( Lanciotti et al. 2008 ). The RT-qPCR conditions were: 50ºC for 5 min, 95ºC for 20 s, and 45 cycles at 95ºC for 3 s, and 60ºC for 30 s with fluorescence acquisition. For all RT-qPCR assays, the MS2 RNA bacteriophage was introduced prior to RNA extraction in order to track false-negative reactions due to PCR inhibition, as described previously ( Naveca et al. 2017 ).

RT-qPCR reactions were analysed by Ct values being Ct ≤ 35 positive for ZIKV RNA presence. Similar studies have considered Ct values ≤ 36 ( Smartt et al. 2017 ) and ≤ 38 ( Ferreira-de-Brito et al. 2016 , Burkhalter & Savage 2017 ) as positive for ZIKV presence in mosquitoes. The Ct is the qPCR cycle where the reporter signal crosses the background fluorescence, and specific amplification is detected. Moreover, the Ct values are inversely linked to the initial quantity of target nucleic acid and can be used to measure its relative concentration in the reaction. Although our experiments were not designed to estimate quantitative data, the obtained Ct values were used as an indirect approach to evaluate viral replication.

We did not evaluate the sensitivity of this assay in vectors, but Lanciotti et al. (2008) reported a sensitivity of 25 copies per reaction. This assay employs a Taqman probe, together with primers specifically designed to ZIKV, which enhances specificity. Furthermore, we used a ZIKV isolate that was previously submitted to nucleotide sequencing that confirms the viral species and genotype.

*Data analysis* - Data were analysed using StepOne V2.3 software (Applied Biosystems). Infection rates were calculated by dividing the number of positive mosquitoes for ZIKV RNA by the number of mosquitoes tested for ZIKV infection. A Student t-test was used to compare the intra-experimental differences between the two biological replicates.

*Ethics statement* - Mosquito field collections were approved by SisBio (Sistema de Autorização e Informação em Biodiversidade - Permission and Information in Biodiversity System) number 12186. This study was approved by the Brazilian National Ethics Committee (CONEP, 3726).

## RESULTS

**Fig. 2:**
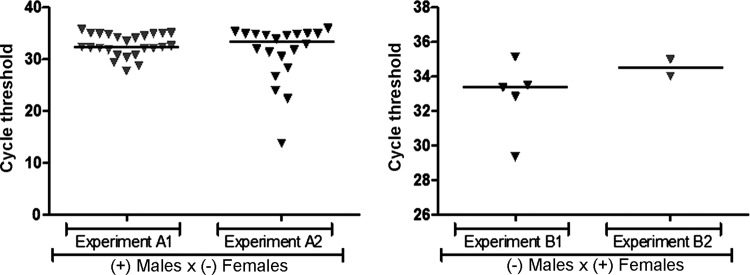
: real-time reverse transcription polymerase chain reaction cycle threshold (Ct) values for *Aedes aegypti* mosquitoes infected with Zika virus (ZIKV) following copulation with infected mates. (A) Negative females that copulated with males infected with ZIKV by microinjection; (B) negative males that copulated with females infected with ZIKV orally.

*Venereal transmission of ZIKV by Ae. aegypti males infected via intrathoracic microinjection* - To determine whether infected *Ae. aegypti* males can transmit ZIKV to uninfected females, 100 adult females were made available for copulation with infected males. Ct values ranged from 13 to 35 in females after mating with intrathoracically infected males ( Fig. 2A ). The mean infection rate in females was 45%, with 40% infected in the first experiment and 50% infected in the replicate (t-test, p = 0.21) ( Table ).

**TABLE:** Infection rates in *Aedes aegypti* mosquitoes infected with Zika virus (ZIKV) following copulation with infected mates

Experiments	Copula	N	Infection rate (%)	Mean infection rate (%)
A1	(+) Males X Females (-)	50	40	45
A2	(+) Males X Females (-)	50	50	
B1	(+) Females X Males (-)	10	50	35
B2	(+) Females X Males (-)	10	20	

(+): ZIKV infected; (-): ZIKV free; N: number of mosquitoes tested for ZIKV infection.

*Venereal transmission of ZIKV by orally infected Ae. aegypti females* - To determine whether infected *Ae. aegypti* females can transmit ZIKV to uninfected males, 20 surviving males (10 from each experimental replicate) were tested for ZIKV transmitted venereally by orally infected females. Ct values ranged from 29 to 35 in males mated with orally infected females ( Fig. 2B ). The mean infection rate in males was 35%, with 50% infected in the first experiment and 20% infected in the second experiment (t-test, p = 0.34).

## DISCUSSION

To complete the life-cycle in nature, the virus must replicate in a mosquito’s tissues. Virus particles then spread throughout the mosquito’s body, and once they reach the salivary glands, they can be transmitted to vertebrate hosts. Some studies have been conducted to ascertain the steps involved in the replication process of DENV, a closely related *Flavivirus* ( Pang et al. 2001 , Salazar et al. 2007 ). In this process, DENV-2 spreads from the midgut at 2 dpi, it disseminates to the salivary glands and other organs at 4 dpi, and it can still be detected at up to 21 dpi ( Salazar et al. 2007 ). Few details on the ZIKV replication process in *Ae. aegypti* are known. This mosquito species has been susceptible to ZIKV ( Li et al. 2012 , Chouin-Carneiro et al. 2016 , Guedes et al. 2017 ). After three or four days of ZIKV challenge, the infection was detected in the midgut, and by seven and 14 days, it is detected in salivary glands. However, the infection rates may change according to the combination of the mosquito population and virus strain. Decreased viral titres were observed by day 14 in the midgut, but ZIKV RNA was detected in the saliva indicating that virus replication occurs in salivary glands during the late phase of infection, suggesting viral transmission ( Guedes et al. 2017 ).

The focus of our work is to demonstrate the venereal transmission of ZIKV in *Ae. aegypti* mosquitoes. Dissemination or transmission rates were not measured. However, as discussed by Li et al. (2012) , the proliferation and dissemination of ZIKV in *Ae. aegypti* is systemic, being possible to find the virus in the midgut, salivary glands, and other tissues such as haemocytes, ganglion, and fat bodies at three-five days after challenge. In addition, Brazilian *Ae. aegypti* is extremely susceptible to ZIKV, with high infection rates found in the midgut and salivary glands (Ferreira-de-Brito et al. 216, Costa-da-Silva et al. 2017 , Guedes et al. 2017 ). Further, in our work, the short time of four-five days was enough to spread the virus in the body of the mosquito, and four-five days of mating were enough for venereal transmission to occur. Higher mating time was not necessary to guarantee venereal transmission because females are inseminated only once in their life ( Lea 1968 ).

Until now, there has been no strong evidence that ZIKV can be transmitted sexually among mosquitoes. However, it is known that female mosquitoes can become infected with arboviruses during haematophagy, and that females can then vertically transmit viruses to their eggs. Vertical transmission of ZIKV has been observed in experimentally infected *Ae. aegypti* specimens ( Thangamani et al. 2016 , Ciota et al. 2017 ). The detection of ZIKV in *Ae. furcifer* males ( Diallo et al. 2014 ) and *Ae. aegypti* males ( Ferreira-de-Brito et al. 2016 ) suggests, but does not prove, that vertical and/or venereal transmission of Zika can occur in these two species.

Our findings showed the presence of ZIKV RNA in previously uninfected mosquitoes of both sexes following copulation with ZIKV-infected mates. These data strongly support the possibility that ZIKV is transmitted in the sexual fluids of mating mosquitoes. This poses a concern to public health because the venereal transmission of Zika among *Ae. aegypti* mosquitoes could potentially increase mosquito infection rates and thereby increase the spread of the virus. Furthermore, if venereal infection occurs in natural mosquito populations, this mode of transmission may be an important mechanism of ZIKV maintenance in nature.

In this study, male mosquitoes were infected with ZIKV via intrathoracic microinjection and transmitted to females during copulation. Additionally, female mosquitoes were orally infected with ZIKV and transmitted to males during copulation. This result indicates that ZIKV systemically disseminates throughout the mosquito body, as it does in other arboviruses from the *Flaviviridae* family. This study shows that ZIKV infection of *Ae. aegypti* mosquitoes occurs not only during blood feeding on infected vertebrates, but also during copulation. To the best of our knowledge, this is the first strong evidence that ZIKV can be transmitted venereally among *Ae. aegypti* mosquitoes.

Several authors speculate that vector to vector transmission is capable of maintaining an arbovirus circulation between interepidemic periods, when epidemiological factors, such as the drop of viral density and the reduction of vertebrate-susceptible hosts, decreases a specific arboviral circulation. Thus, we believe that venereal transmission as well as natural transovarian transmission may play an important role aiding the establishment of endemic arboviral cycles.

Experts at the WHO have recently declared that “ZIKV and its associated consequences remain a significant and enduring public health challenge that require intense action, but ZIKV no longer represents a Public Health Emergency of International Concern” (WHO 2016b). In the global context, the dissemination of *Aedes* vectors is of such scope that future outbreaks of ZIKV and other flaviviruses will be difficult to foresee. Moreover, in the absence of a vaccine, our ability to block the spread of ZIKV relies solely on vector control measures. Therefore, studies that increase our understanding of viral-host biological interactions are of great importance and should be encouraged.
